# Interspike intervals under the constraint of linear synaptic integration and background synaptic activity

**DOI:** 10.1186/1471-2202-14-S1-P239

**Published:** 2013-07-08

**Authors:** Danielle Morel, William B Levy

**Affiliations:** 1 Department of Physics, Stetson University, DeLand, FL 32723, USA; 2 Departments of Neurosurgery and of Psychology, University of Virginia, Charlottesville, VA 22908, USA

## 

Previous work [1] using a one-compartment steady-state computer model of a neuron shows how combinations of two active dendritic conductances can produce a linear dendritic response over a voltage range of ca. 15 to 22 mV. Here we extend this linearization issue to a dynamic, non-steady state situation for a neuron model with a soma, dendrite and distal compartment. It is known that synaptically-induced voltage pulses that originate in distal compartments will be both attenuated and broader by the time they reach the soma due to the passive cable properties of neurons. Additionally, neurons in intact brains are not isolated but are under constant synaptic bombardment. While a single synaptic event will not significantly affect the cell properties, thousands of such events will significantly change the integrative properties of neurons [2]. Due to the reduction in membrane resistance caused by synaptic activity, attenuation of individual voltage pulses is even stronger in the presence of background synaptic activity. This study shows that the presence of active conductances distributed along the cell membrane can mitigate some of the effects of electrical filtering on voltage pulses propagating to the soma, even in the presence of background synaptic activity.

We consider three uniformly distributed conductances found in pyramidal cells of the forebrain: persistent sodium current (*I_NaP_*) [3,4], hyperpolarization-activated mixed cation current (*I_h_*) [5], and A-type potassium current (*I_KA_*) [6]. Added to an otherwise passive cell, select combinations of these voltage-activated ion channels can generate linear synaptic integration over the entire interspike interval (ISI) membrane potential range, that is from rest (here -72 mV) to threshold (here roughly -45 mV). In the presence of various levels of background synaptic activity, the ratio of these active conductances changes and contributes to the inverse ISI effect. Under the constraint of linear synaptic integration, the length of the ISI is seen to be inversely related to the intensity of the background synaptic activity. This inverse relationship results from a decrease of the effective time constant of the cell by increased levels of membrane conductance induced by the combination of synaptic activity and active conductances.
